# Supplementary material for: Estimating total human deaths from rabies: A statistical framework leveraging reports of rabies transmission from deceased organ and tissue donors
Source: PLoS Negl Trop Dis. 2026 Sep 21;20(9):e0014731. doi: 10.1371/journal.pntd.0014731 (PMC13592719; doi:10.1371/journal.pntd.0014731)
Supplement: Supplementary file 6 [file pntd.0014731.s005.zip › Result_A/Kazakhstan/Figure_S3_Kazakhstan.pdf]

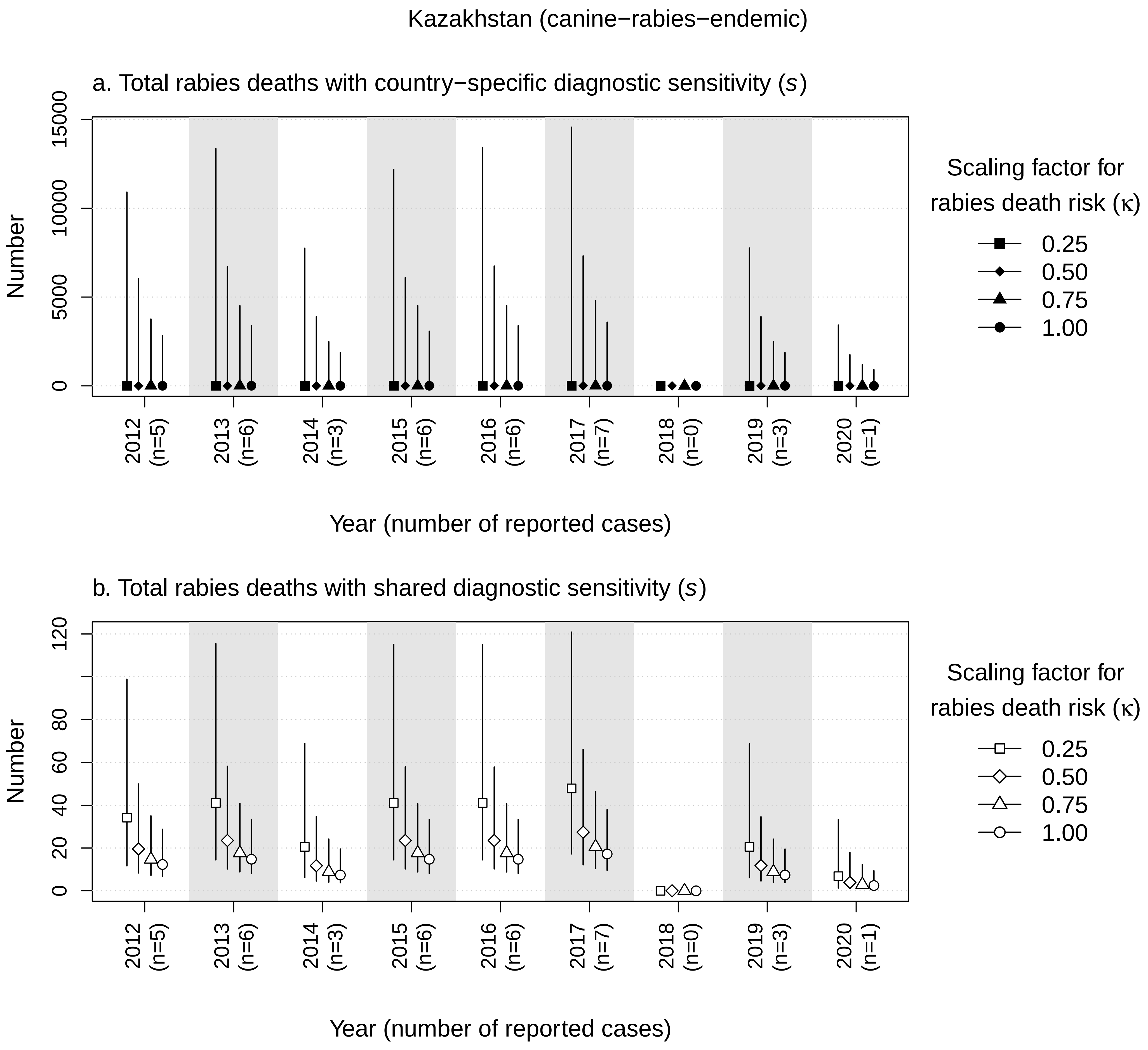

Kazakhstan (canine−rabies−endemic)
a. Total rabies deaths with country−specific diagnostic sensitivity (s)
Number
15000
10000
5000
0
2012 (n=5)
2013 (n=6)
2014 (n=3)
2015 (n=6)
2016 (n=6)
2017 (n=7)
2018 (n=0)
2019 (n=3)
2020 (n=1)
Year (number of reported cases)
Scaling factor for rabies death risk (κ)
0.25
0.50
0.75
1.00
b. Total rabies deaths with shared diagnostic sensitivity (s)
Number
120
80
60
40
20
0
2012 (n=5)
2013 (n=6)
2014 (n=3)
2015 (n=6)
2016 (n=6)
2017 (n=7)
2018 (n=0)
2019 (n=3)
2020 (n=1)
Year (number of reported cases)
Scaling factor for rabies death risk (κ)
0.25
0.50
0.75
1.00
